# Breast cancer metastases to the stomach with endoscopic submucosal dissection: a case report and literature study

**DOI:** 10.3389/fonc.2025.1573163

**Published:** 2025-05-22

**Authors:** Qingmiao Zhao, Lizhou Dou, Xiaolong Feng, Guiqi Wang, Shun He

**Affiliations:** ^1^ Department of Endoscopy, National Cancer Center/National Clinical Research Center for Cancer/Cancer Hospital, Chinese Academy of Medical Sciences and Peking Union Medical College, Beijing, China; ^2^ Department of Pathology, National Cancer Center/National Clinical Research Center for Cancer/Cancer Hospital, Chinese Academy of Medical Sciences and Peking Union Medical College, Beijing, China

**Keywords:** case report, breast cancer, gastric metastasis, endoscopic submucosal dissection, immunohistochemistry

## Abstract

Breast cancer (BC) is the second leading cause of cancer-related death in women, with mortality primarily associated with metastasis. Although gastric metastasis is rare, there are still case reports in the literature. Clinical symptoms of gastric metastasis from BC are often nonspecific, and endoscopic presentations are heterogeneous. Distinguishing between primary and metastatic tumors is important and challenging for the endoscopist. Herein, we report a case of gastric metastasis occurring 4 years after BC surgery. The timeline is as follows. In 2019, the 60-year-old woman received neoadjuvant chemotherapy followed by left breast radical surgery and endocrine therapy with targeted treatment. In 2022 (3 years post-surgery), left axillary metastasis was diagnosed, requiring excision of a skin mass. In 2023 (4 years post-surgery), she presented with upper abdominal pain and acid reflux, and gastroscopy revealed a superficial flat lesion in the gastric antrum. Immunohistochemical (IHC) staining suggested the possibility of poorly differentiated adenocarcinoma. Contrast-enhanced computed tomography (CT) revealed focal abnormal enhancement of the gastric antrum without evidence of distant metastasis. Since endoscopic, imaging, and IHC staining findings did not clearly suggest BC metastasis, and considering the patient’s history of BC and overall condition, she underwent endoscopic submucosal dissection (ESD)-a procedure performed with both diagnostic and therapeutic intent. The postoperative pathology revealed metastatic invasive lobular carcinoma (ILC) of the breast. The ESD not only clarified the patients’ diagnosis but also avoided unnecessary surgery for the patient. The patient remains alive under maintenance therapy. In summary, our case highlights the role of ESD, which was performed with both diagnostic and therapeutic intent—in managing gastric metastasis from BC, while underscoring the necessity of regular endoscopic surveillance post-mastectomy.

## Introduction

Breast cancer (BC) most frequently metastasizes to soft tissues, brain, liver, bones, and lungs, while gastrointestinal (GI) tract involvement remains uncommon. Follow-up and autopsy studies have estimated the incidence of gastric metastasis to range from 0.2% to 0.7% (1). Gastric metastasis from BC tends to occur several years after the initial diagnosis. The clinical symptoms are nonspecific and diverse, ranging from asymptomatic cases to those with severe GI symptoms, which complicates and challenges the diagnostic process (2, 3). Invasive lobular carcinoma (ILC) is more likely to metastasize to the GI than invasive ductal carcinoma (IDC) (4). A study by Almubarak MM et al. found that 97% of GI metastases from BC originated from the ILC (5, 6). The primary treatment is systemic therapy, tailored to the individual’s general medical condition and the tumor’s biological hormonal receptor and HER2 status (6). We report a case of gastric metastasis from BC, highlighting the experience in differentiating between metastatic and primary tumors, as well as the benefits of ESD, combining diagnostic and therapeutic intent, in patients with a history of BC who present with lesions resembling early gastric cancer. We believe that in patients with a history of BC presenting with symptoms such as anorexia, nausea, vomiting, stomach pain, and gastric discomfort, it is essential to consider whether these symptoms are due to metastasis from the primary tumor or treatment-related discomfort. However, the possibility of a second primary tumor should not be overlooked. Timely detailed gastroscopy, imaging study, and immunohistochemical (IHC) staining comparison are crucial for accurate diagnosis. This study adheres to the CARE case report guidelines (7).

## Case report

A 60-year-old woman denied a history of chronic diseases such as hypertension, heart disease, and diabetes. Her father and aunt were patients with lung cancer, and she denies a family history of other tumor diseases. In July 2019, the patient presented with a palpable right breast mass and was subsequently diagnosed with stage IV ILC (T4N3M1) accompanied by axillary lymph node and skeletal metastases. She underwent four cycles of neoadjuvant chemotherapy with paclitaxel and doxorubicin. Two months later, in September 2019, she underwent modified radical mastectomy of the right breast plus axillary lymph node dissection. The postoperative pathology revealed ILC of the breast (2.2 cm in diameter), with no special findings at the nipple and base margins, and lymph node metastasis of 14/14. IHC staining showed estrogen receptor (ER)-positive, progesterone receptor (PR)-positive, human epidermal growth factor-2 (HER2)-negative, and Ki-67 (10%). Postoperatively, she was treated with a combination of fulvestrant and palbociclib, with regular follow-ups.

Three years later, in June 2022, she noticed a mass in the skin of her left axilla and underwent excision. The postoperative pathology confirmed metastasis of ILC. IHC staining showed ER-positive, PR-positive, HER2-positive, Ki-67 (30%), and P120 (cytoplasmic+). One month later, Positron Emission Tomography/Computed Tomography (PET-CT) showed no other abnormal metabolic increases. Concurrently, the treatment plan was adjusted to a combination of exemestane and chidamide, with chidamide at 30 mg twice weekly and exemestane at 25 mg once daily, both administered orally.

**Figure 1:**
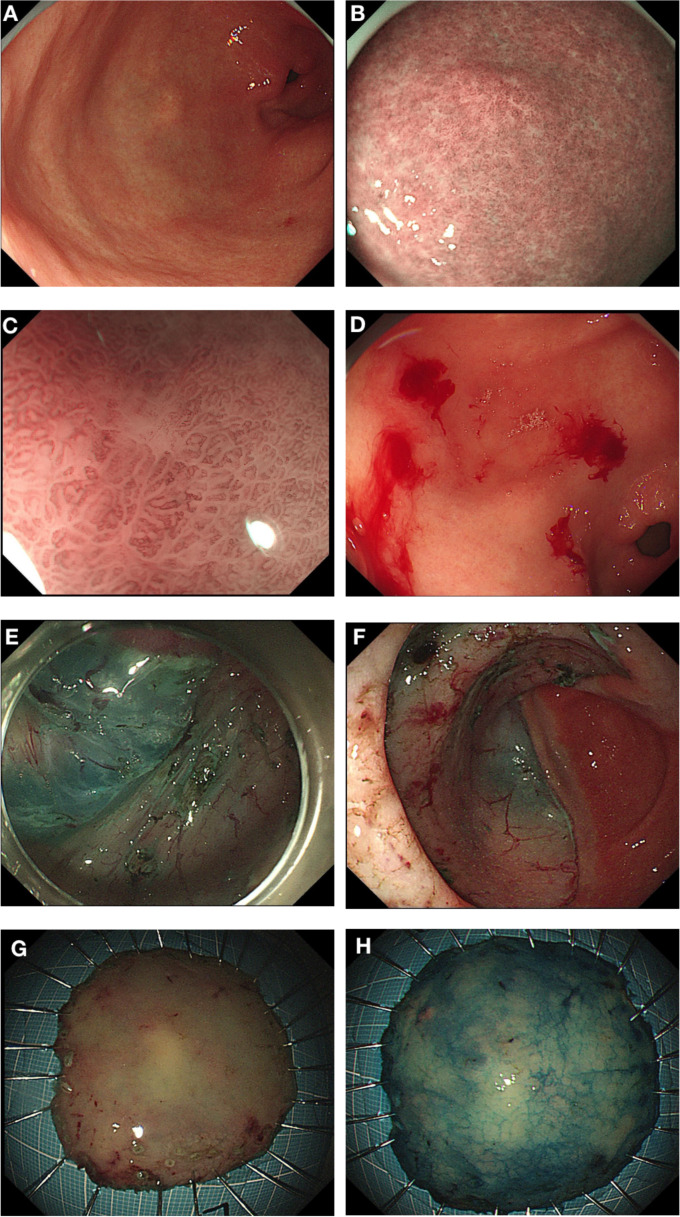
Endoscopic Presentations. **(A)** A 0-IIb type superficial flat lesion on the anterior wall of the gastric antrum (0.5*0.5 cm, about 60 cm from the incisors), with a faded color. **(B, C)** Magnifying endoscopy with narrow-band imaging (NBI) showed DL (-), with slightly irregular MV and MS. **(D)** Negative biopsies from the periphery of the lesion to delineate the extent of the tumor. **(E)** Dissect along the submucosa. **(F)** The dissected base. **(G, H)** ESD specimen.

**Figure 2:**
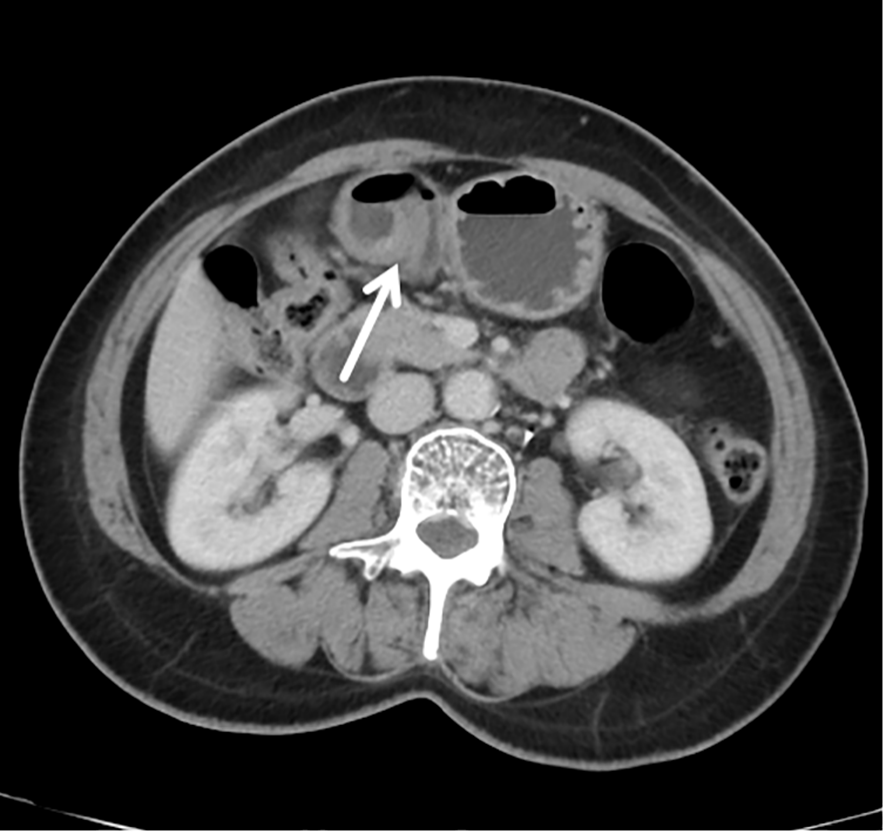
Contrast-enhanced computed tomography (CT) revealed focal abnormal enhancement of the gastric antrum without other obvious metastasis.

**Figure 3:**
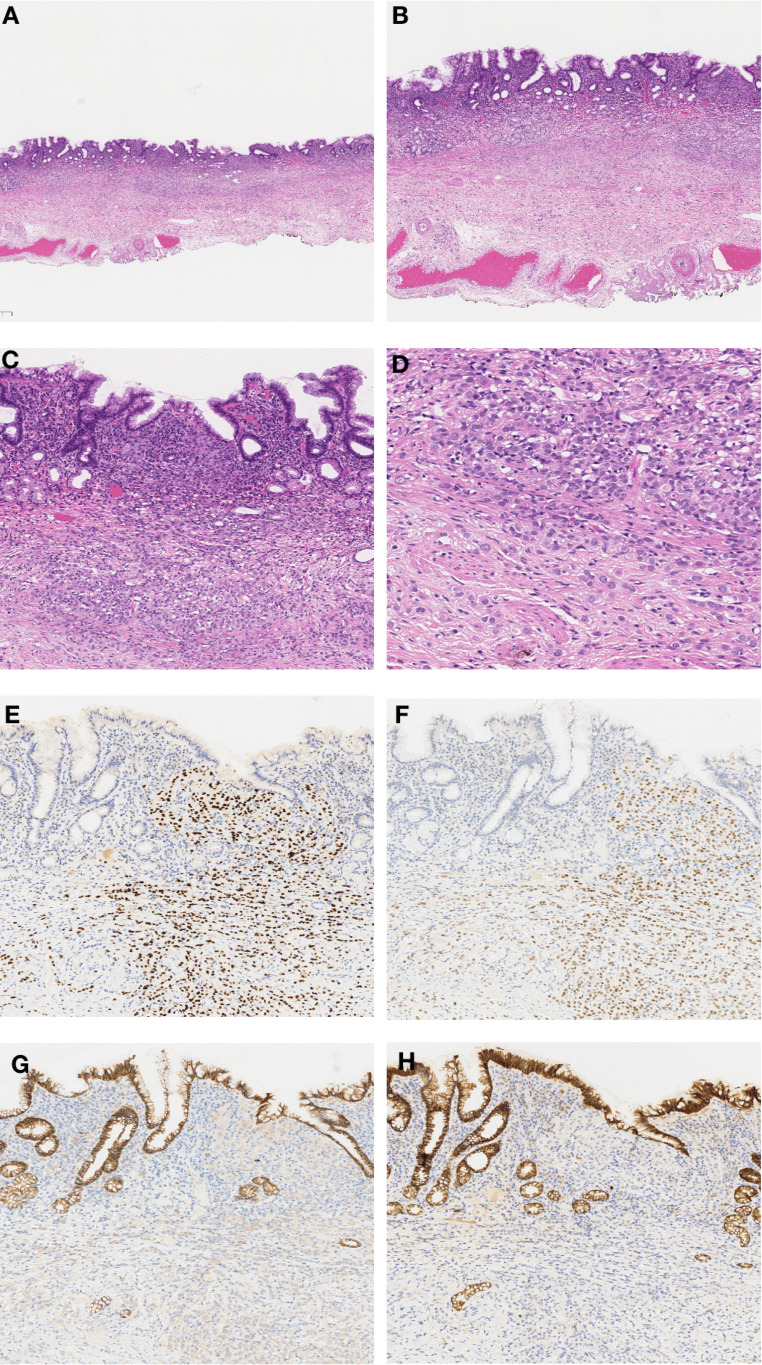
Histopathological and Immunohistochemical Findings. **(A)** ESD specimen showing localized widening and dilatation of the gastric foveolar base (×20). **(B)** Diffuse cellular infiltration in the widened foveolar area (×40). **(C)** Tumor cell infiltration from lamina propria to submucosa (×100). **(D)** Tumor cells with disorganized distribution, some with mucin or clear cytoplasm; fine chromatin and prominent nucleoli (×200). **(E)** Strong GATA3 positivity (×100). **(F)** ER positivity in 80% of tumor cells (×100). **(G)** Weak cytoplasmic P120 positivity (×100). **(H)** Negative E-cadherin staining (×100).

In September 2022, due to complaints of upper abdominal pain and acid reflux, she underwent endoscopic examination, which revealed a 0.5*0.5 cm, 0-IIb type superficial flat lesion on the anterior wall of the gastric antrum (about 60 cm from the incisors), with a faded color (**Figure 1A**). Magnifying endoscopy with narrow-band imaging (NBI) revealed an absent demarcation line (DL), with slightly irregular microvascular (MV) and microsurface (MS) patterns (**Figures 1B, C**). Simultaneously, given that the patient had undergone endoscopy and biopsy at another hospital, we obtained negative biopsies from the periphery of the lesion to delineate the extent of the tumor (**Figure 1D**). The pathology consultation revealed a few atypical cells within the gastric mucosa of the antrum, and in combination with immunohistochemical staining, a poorly differentiated adenocarcinoma was considered. Therefore, we diagnosed this lesion as early gastric cancer, which was eligible for endoscopic resection. Six months earlier, the patient had undergone PET-CT after resection of a metastatic mass in the left axilla, showed no other abnormal metabolic increases. Preoperative CT depicted focal abnormal enhancement of the gastric antrum without other obvious metastasis (**Figure 2**). In February 2023, ESD was performed (**Figures 1E-H**). Histopathological examination of the ESD specimen revealed that the tumor cells infiltration from lamina propria to submucosa (**Figures 3A-D**). IHC findings shows strong positivity for GATA3 (**Figure 3E**), 80% positivity for ER (**Figure 3F**), weak positivity for cytoplasmic P120 (**Figure 3G**), and negativity for E-cadherin (**Figure 3H**). Finally, this case was diagnosed as BC metastases to stomach. Postoperatively, targeted therapy for malignant tumors was administered, with a combination of chidamide tablets and exemestane tablets, chidamide tablets at 30 mg multiple times weekly orally, and exemestane tablets at 25 mg once daily. The patient is alive, receiving maintenance therapy. Written informed consent was obtained from the patient for the publication of this case report and any accompanying images.

## Discussion

### Epidemiology

BC is the most common cancer in women and the second leading cause of cancer-related deaths, following lung and bronchial cancer (8). The mortality rate is primarily associated with metastasis (9). BC commonly metastasizes to soft tissues, brain, liver, bones, and lungs, with gastrointestinal (GI) tract metastasis being relatively rare. Montagna et al. reported that among 2,588 patients with metastatic BC, 1.55% had GI metastasis (10). McLemore et al. found that only 0.34% of 12,001 patients with metastatic BC had GI metastasis (11). The incidence of gastric metastasis from BC, as reported in clinical studies, ranges from 0.2% to 0.7%, while autopsy studies estimate it to be between 0.8% and 18%. This discrepancy highlights the importance and limitations of clinical diagnosis (1). Most cases originate from ILC (1). Luminal type A or B breast cancer tends to commonly metastasize to the stomach. Furthermore, ILC metastasis to the stomach is more frequent when considering pathological type (12). The most common site of GI metastasis from BC is the stomach (60%), followed by the esophagus (12%), colon (11%), small intestine (8%), rectum (7%), oropharynx (1%), and anus (1%) (13).

### Clinical symptoms

Patients with gastric metastasis from BC typically present with GI symptoms, with a minority being diagnosed incidentally during routine follow-up or physical examination (14). Common symptoms include anorexia, dysphagia, early satiety, postprandial bloating, epigastric pain, melena, nausea, and vomiting (1). These symptoms can sometimes mimic side effects of chemotherapy or other medications, making clinical diagnosis more challenging. This nonspecific presentation underscores the need for improved endoscopic screening and surveillance strategies, particularly in patients with a known history of BC presenting with new GI symptoms. Endoscopic evaluation and biopsy, followed by IHC staining, are integral to the diagnostic workup, as they play a crucial role in confirming the breast origin of the metastatic lesions (15, 16). Interestingly, Rech et al. 17 reported a case of gastric metastasis from BC presenting with a paraneoplastic rheumatic syndrome (PMR-like), such as arthralgia, weight loss, moderate anemia and elevated acute-phase markers. The patient was treated with prednisone and chemotherapy, which greatly improved the symptoms. This literature provides new perspectives on the clinical symptoms of gastric metastasis from BC (17).

### Endoscopic presentations

Endoscopic appearances are heterogeneous, ranging from benign-appearing lesions (such as gastritis) to diffuse or ulcerated tumo.rs (mimicking primary gastric carcinoma or lymphoma) (12). Three main types of lesions have been reported: localized tumor deposition (18%), diffuse infiltration (i.e., linitis plastica type or gastritis) (57%-73%), and external compression (25%). Diffuse infiltration of the gastric wall is a representative form of gastric metastasis from BC, known as the linitis plastica type (18). White light endoscopy may reveal enlarged mucosal folds, erosions, or polypoid lesions. However, metastasis can also present as localized lesions in the stomach, appearing as flat elevated, erosive, ulcerative, or polypoid lesions, which may mimic early-stage gastric cancer (18, 19). Since it is difficult to distinguish between primary gastric cancer and metastatic gastric cancer using hematoxylin and eosin staining alone, it is essential to perform detailed IHC examinations on biopsy specimens, which is crucial for diagnosis (20). The case reported herein presented as a superficial flat lesion, with endoscopic and histopathological findings closely resembling poorly differentiated early gastric adenocarcinoma. If IHC had not been performed on the ESD specimen, the patient would have been diagnosed with primary gastric cancer. In many studies, patients were initially diagnosed with primary gastric cancer and only later, after gastrectomy, were diagnosed with metastatic BC, leading to unnecessary surgical intervention (21, 22).

### Diagnosis

There is typically a 5- to 7-year interval between the initial diagnosis of BC and the development of gastric metastasis (23). The diagnosis of gastric metastasis from BC is challenging due to the nonspecific and mimicking nature of clinical symptoms, the heterogeneity of endoscopic appearances, and the difficulty in differentiating between primary and metastatic gastric cancer. When faced with patients with a history of BC, endoscopists should carefully inquire about clinical symptoms, perform detailed endoscopic examinations, and inform pathologists to conduct thorough IHC comparisons, managing these patients with a comprehensive and multidisciplinary approach. Xu et al. reported the largest case series (n=78) on gastric metastasis from BC to date, describing abdominal pain as the most common symptom (75.6%), which is consistent with the present case. Notably, while 27 patients (34.6%) developed metastasis in other organs prior to gastric involvement, 49 patients (62.8%) presented with synchronous metastases, usually in bone (50%), lung (12.2%), and liver (20.4%). This pattern underscores the clinical necessity for comprehensive multi-organ evaluation during the diagnostic workup of suspected gastric metastasis (24). The primary molecular markers currently used to differentiate between primary and metastatic gastric tumors include ER, PR, HER2, GATA3. GATA3 and ER positivity can suggest metastatic breast cancer, which are negative in primary gastric cancer. E-cadherin and cytoplasmic P120 can be used to distinguish between ILC and IDC. E-cadherin negativity combined with cytoplasmic P120 positivity is indicative of ILC. Studies suggest that approximately 90% of ILC exhibit a lack of E-cadherin, which can promote tumor metastasis and is a critical factor in BC dissemination (25).

### Treatment and prognosis

Data on the treatment of gastric metastasis from BC are limited. The primary treatment modality is systemic therapy tailored to the tumor’s receptor profile, with most patients receiving chemotherapy or a combination of chemotherapy and endocrine therapy, and radiation therapy being rarely used (13, 19). Surgical intervention is considered in specific scenarios such as obstruction or bleeding. To date, there have been two case reports of ESD treatment for gastric metastasis from BC. In October 2016, Masahide Kita et al. reported a 52-year-old female with a history of ILC who underwent ESD treatment and subsequently received anastrozole. However, multiple metastatic tumors in the gastric body were detected 40 months after endoscopic treatment (18). In August 2023, Kosuke Tanaka et al. reported a 79-year-old female with a history of ILC who underwent ESD treatment. Within one month after ESD, CT revealed axillary lymphadenopathy, suggesting metastatic BC. The patient received chemotherapy but died 20 months later (19). The prognosis for patients with gastric metastasis from BC is generally poor, closely related to the site and extent of metastatic disease and the hormonal receptor status of the tumor. The estimated median survival rate after diagnosis of gastric metastasis is 10–28 months, with further reduction in patients with multi-organ metastasis (1).

## Conclusion

Gastric metastasis from BC is rare in clinical practice. To date, the literature on gastric metastasis from BC remains incomplete, with the majority consisting of case reports and reviews, and few small retrospective studies. Clinical symptoms are nonspecific and often mimic other primary gastrointestinal diseases or side effects of breast cancer drug treatments. Endoscopic findings are heterogeneous, ranging from benign-appearing lesions (such as gastritis) to diffuse or ulcerated tumors (resembling primary gastric cancer or lymphoma). This presents many challenges for clinical diagnosis. This case underscores the imperative for multidisciplinary collaboration between endoscopists, pathologists, and oncologists in the diagnostic algorithm of BC survivors with gastrointestinal symptoms. When endoscopists identify a suspicious gastric lesion during endoscopy, the possibility of metastasis should be considered, and the pathologist should be informed of this possibility. Early biopsy should be performed to obtain a histopathological diagnosis, distinguishing between primary GI disease, primary GI cancer, and metastatic GI disease. When histopathological confirmation proves inconclusive through conventional biopsy, ESD emerges as both a diagnostic and therapeutic modality, particularly in patients with BC history presenting with indeterminate gastric lesions.

## Data Availability

The raw data supporting the conclusions of this article will be made available by the authors, without undue reservation.
